# High blood levels of lead in children aged 6-36 months in Kathmandu Valley, Nepal: A cross-sectional study of associated factors

**DOI:** 10.1371/journal.pone.0179233

**Published:** 2017-06-12

**Authors:** Meghnath Dhimal, Khem Bahadur Karki, Krishna Kumar Aryal, Bimala Dhimal, Hari Datt Joshi, Sajan Puri, Achyut Raj Pandey, Purushotam Dhakal, Arun Kumar Sharma, Ganendra Bhakta Raya, Imran Ansari, David A. Groneberg, Ruth Müller, Ulrich Kuch

**Affiliations:** 1 Nepal Health Research Council (NHRC), Ramshah Path, Kathmandu, Nepal; 2 Department of Pediatrics, Tribhuvan University Teaching Hospital, Kathmandu, Nepal; 3 Siddhi Memorial Hospital, Bhaktapur, Nepal; 4 Department of Pediatrics, Patan Academy of Health Sciences, Lalitpur, Nepal; 5 Institute of Occupational Medicine, Social Medicine and Environmental Medicine, Goethe University, Frankfurt am Main, Germany; TNO, NETHERLANDS

## Abstract

Young children are at greatest risk of exposure to lead and its effects. Although lead is one of the most widely used elements with known health hazard, there is little data on the blood lead level (BLL) of children in the Kathmandu Valley. Thus, this study aimed to assess factors associated with high BLL in children who were 6–36 months of age and resided in the Kathmandu Valley. In this hospital-based cross-sectional study 6–36 month-old children visiting the Paediatrics Outpatient Department of Tribhuvan University Teaching Hospital, Patan Hospital, and Siddhi Memorial Hospital were enrolled. All three hospitals are located in different areas inside the Kathmandu Valley. Written informed consent was obtained from the parents, and exposure data were collected using a structured questionnaire. Portable Anodic Stripping Voltammetry (ASV) was used to determine BLLs in children. Data were analyzed using SPSS version 16. Of 312 children enrolled in the study, 64.4% had BLLs ≥5μg/dl. A significant association was found between BLL and exposure to enamel paints in the household in the form of painting materials used in different parts of the house like walls, windows and doors (*p* = 0.001). Furthermore, multivariate analyses showed that BLLs were 4.5 times higher in children playing with dirt and dust (*p* = 0.006) and that children belonging to the community of lower caste/ethnicity groups had significantly higher BLLs compared to those from the upper caste groups (*p* = 0.02). Our study demonstrated that children living in households that have used enamel paints, children belonging to lower caste/ethnic groups, and children frequently playing with dirt and dust had significantly higher BLLs. The results of this study highlight the importance of policy decisions to limit environmental lead contamination, and to roll out awareness building measures designed to limit lead exposure and break the poverty cycle associated with chronic lead poisoning.

## Introduction

Lead is a non-ferrous metal that is widely used in a large variety of industries and consumer products. Although it is a non-essential element for the human body, lead is readily taken up via inhalation of polluted air and ingestion of contaminated water, food, soil, dust or paint, and accumulated in blood, bone, and soft tissues [1,2]. Acute lead poisoning can result in multiple organ dysfunction, but chronic lead poisoning is equally worrisome and much more common [2,3]. Being a cumulative toxicant, lead affects multiple body systems and is particularly harmful to young children whose bodies absorb the toxic metal at higher rates than adults [2,3]. Lead exposure in early childhood leads to identifiable deficits in cognitive development and these effects are largely irreversible [3–5]. Children who have been exposed to lead are more likely to have lower intelligence quotients, long-term health problems, be diagnosed with attention deficit hyperactivity disorder, and exhibit aggressive behaviour [3,4,6]. Childhood lead exposure is estimated to contribute to about 600,000 new cases of children developing intellectual disabilities and 143,000 deaths each year with the highest burden in developing regions [2]. In addition, the International Agency for Research on Cancer (IARC) has classified lead as a probable carcinogen [7].

No safe limits for the blood lead level (BLL) have been identified and even at low levels, lead has been shown to affect the intelligence quotient, attention span and academic performance in children [4,6,8]. However, since 2012, the U.S. Centers for Disease Control and Prevention (CDC) have recommended BLLs >5 μg/dl as a reference level for intervention based on the U.S. National Health and Nutrition Examination Survey [9]. This threshold level was redefined for activating targeted interventions for children in order to reduce their continued exposure to lead and the resulting health effects [9].

In the past, lead was commonly used in gasoline as an anti-knocking agent. However, the gasoline that is currently used in Nepal is unleaded. Apart from gasoline, lead is also widely used in electronics, ceramics, crystal glass, lead-acid batteries, cables and paint. A rising demand and continued use of lead in a wide range of industrial and consumer products have greatly increased its circulation in air, water and soil, and consequently the exposition of a vast number of people worldwide [3]. Lead is widely used in lead-based paint; lead-acid batteries; lead plates, sheets, strip foil, lead tubes, pipes and fittings; cable sheathing and alloys (lead alloys, unwrought). In household settings, the risk of lead exposure from lead acid batteries and lead-based paint is of greatest concern [2]. For example, it has been reported that 71% of all paint products sold in Nepal had lead levels above 90 ppm [10]. Another study conducted by Leaders Nepal found lead concentrations up to 200,000 ppm (20% lead by weight) in some brands of paints that are commonly available in Nepalese markets indicating serious population level exposure [11].

We also expect that the use of enamel paints is higher in urban areas. Similarly, small children under three years are most likely to be exposed to environmental lead because of their play behavior and developmental characteristics. In this context, the present study was conducted to assess the BLL of children aged 6–36 months living in the Kathmandu Valley of Nepal which comprises the biggest urban area of Nepal, and to determine the factors that were associated with higher BLLs in these children.

## Materials and methods

### Study site and its justification

This study was conducted in the Kathmandu Valley where lead-based paints are more widely used compared to rural areas of Nepal. Three major hospitals in different geographic locations of the Kathmandu Valley participated in the study (Patan Hospital, Tribhuvan University Teaching Hospital, and Siddhi Memorial Hospital). Equal numbers of children of the 6–36 month age group visiting the outpatient departments (OPD) were enrolled from these hospitals.

### Inclusion and exclusion criteria

Children who had been brought to the OPD by their parents for general health checks, immunization, and growth monitoring, were eligible for participation in the study. After a physician’s evaluation, the parents of eligible children were counselled about the BLL study and interested parents were referred to the study team for further information. The study team then explained the objectives as well as potential risks and benefits of the study. Children whose parents provided written informed consent to participate were enrolled in the study. Children who had been admitted indoor (inpatients), those who had been given Ayurvedic medication by their parents, and children with known current accidental exposure to lead sources were excluded. Posters were displayed in the OPD of each hospital about sources of lead in the environment and its negative effects on human health.

### Sampling and sample size

We calculated the sample size using the algorithm suggested by van Belle [12]. We assumed a type I error of 0.05, a type II error of 0.20, standard deviation 6, a non-response rate of 15% and 80% power for calculating the sample size for one sample. From each of three hospitals (Patan Hospital, Tribhuvan University Teaching Hospital, and Siddhi Memorial Hospital) an equal number of children of the 6–36 month age group were enrolled from visitors of the respective OPD. Purposive sampling was done to choose the study subjects on a ‘first come first’ basis. After counseling patients about the objectives of the study, provided by a physician, and excluding some of them as per the exclusion criteria, children were enrolled in our study. During the counseling of parents by the physician, the response rate to participate in the study was approximately 80%. Patients fulfilling the inclusion criteria and whose parents were willing to participate in the study were enrolled in the study until the required sample size was attained.

### Data collection tools and techniques

Data were collected using the following data collection techniques: (i) structured interview questionnaires and (ii) lead concentration measurement in capillary blood. A structured interview questionnaire administered by trained interviewers was used to predict the lead exposure of the children.

The BLL was measured using the Lead Care II Blood Analyzer based on the electrochemical Anodic Stripping Voltammetry (ASV) method. About two drops of blood (50 μl of capillary blood) was obtained by standard finger prick technique, mixed with a reagent, and a portion dispensed onto the single use disposable electrodes (sensors) of the analyzer where the plating and subsequent stripping of lead took place. The BLL was determined within 3 minutes of test. The reportable range of the LeadCare II system is 3.3 to 65 μg/dL. In general, BLL is accepted as the most valid and reliable indicator of recent excessive lead exposure. The ASV device is approved by the WHO and the United States Food and Drug Administration (USFDA) and is commonly used in mobile health units in environmental and occupational health studies. Stanton and Fritsch have shown a good agreement of BLL test results between the Lead Care ASV and Atomic Absorption Spectrometry methods [13]. As a quality control measure, blood collection was conducted after thoroughly cleansing the fingertip with wet tissue paper containing soap and alcohol. Once washed, the cleaned finger was not allowed to come into contact with the other fingers or any surface. Furthermore, it has been found that capillary blood sample collection by fingerstick has very low (<10%) contamination error rates [14,15].

The study questionnaire was designed using a template from a previous study [16] with modifications to meet the objectives of our study. The study questionnaire was prepared in English, translated into Nepali and back translated to ensure consistency with the original version in English. Before using the tool in the actual study, the reconstituted questionnaire was pilot-tested in Siddhi Memorial Hospital and necessary adjustments were made. The data from the pilot test were not used in the main study.

### Data analysis

The data was entered into a database by trained personnel using the Epi Data 3.1 Software (EpiData Association, Denmark). All data files were checked and cleaned by two of the authors before analysis. The data were transferred to, and analysis was performed in SPSS version 16. Both univariate and multivariate analysis were carried out for possible factors associated with elevated BLL among the participating children. We had found that more than 10% of the children had BLL less than 3.3 μg/dL(displayed low in screen) and BLL could not be measured as a continuous dependent variable. Hence, we followed the CDC guideline for elevated blood lead levels (BLLs ≥5 μg/dl) and the blood lead level of the children included in the study was described as low (0–5 μg/dl) and high (≥ 5 μg/dl). The blood lead levels of children were compared using a Chi-square test. Fisher’s exact test was used where more than 20% of the cells had expected cell counts less than five. Information on some variables which were missing were reported as "missing" in analysis. Logistic regression analysis was carried out to identify determinants of the blood lead level of children. Independent variables included in the model were sex of children, age group, ethnicity, education status of mother, education status of father, occupational status of mother, occupational status of father, household painted with enamel paint, school area painted with enamel paint, children sleeping and playing in area with enamel paint, children playing outdoors, children playing with dust and dirt, children playing with batteries, children playing with painted toys and children peeling off paint from walls/windows. A dependent variable introduced to the model was the blood lead level (i.e., “low” vs.”high”). Confounding factors were explored observing the difference between the adjusted odds ratio (aOR) in multivariate analyses and the crude odds ratio (OR) from univariate analyses of a particular independent variable with another independent variable. Determinants having a screening significance of *p* < 0.25 in univariate analysis were selected for multivariate analyses. We tested the multi-collinearity of variables before including them in multivariate analysis. The correlation between these variables was less than 0.5. All *p*-values are two tailed and were considered to be statistically significant at *p* < 0.05.

### Ethics approval and consent to participate

The National Ethical Review Board (ERB) of the Nepal Health Research Council (NHRC) provided ethical clearance for this study. Informed written consent was obtained from the parents of children before their participation in the study. All study questions were asked to parents by study team members in a designated corner of the room away from other patients maintaining full confidentiality.

## Results

Table 1 shows the distribution of children by age and sex groups. Of the total 312 children enrolled in the study, 56.7% were male and 43.3% female.

**Table 1:** Background characteristics of the study population.

Variable	Number (%)
**Age group**	
6–12 months	99 (31.7)
13–24 months	150 (48.1)
25–36 months	63 (20.2)
**Gender**	
Male	177 (56.7)
Female	135 (43.3)
**Ethnicity**	
Upper caste groups	62 (31.2)
Socially disadvantaged caste groups	250 (68.8)
**Fathers’s education**	
Literate	302 (96.8)
Iliterate	10 (3.2)
**Mother’s education**	
Literate	278 (89.1)
Iliterate	33 (10.6)
**Father’s occupation**	
Agriculture	28 (9.1)
Own business	48 (15.6)
Service	91 (29.5)
Student	43 (14.0)
Other	98 (31.8)
**Mother’s occupation**	
Housewife	233 (75.2)
Agriculture	13 (4.2)
Own business	15 (4.8)
Service	27 (8.7)
Other	22 (8.7)
**Children in school**	
Already enrolled in nursery/school	43 (13.7)
Not enrolled in nursery/school	269 (86.3)

### Blood lead levels of the study population

Table 2 shows the BLLs of the study population. The median BLL was 5.8 μg/dl (IQR: 4.3, 9.97). Blood lead levels in excess of 5 μg/dl, the level currently recommended by the U.S. CDC as the threshold for activating targeted interventions, was found in 64.4% of the study population. However, none of the children in our study had BLLs that would have warranted treatment, i.e, chelation therapy as per CDC guidelines.

**Table 2:** Blood lead levels of the study population.

Blood lead level (μg/dl)	Number of children (%)
0–5	111 (35.6)
5–10	124 (39.7)
10–15	37 (11.9)
15–20	15 (4.8)
20–25	12 (3.8)
25–30	6 (1.9)
30–35	2 (0.6)
35–40	3 (1)
40–45	2 (0.6)
0–45	312 (100)

### Factors associated with high blood lead levels

Our study found that 64.3% of the children had BLL ≥5 μg/dl (Table 2). Table 3 shows factors associated with high BLLs. The majority (68.8%) of children with BLL ≥5 μg/dl were from socially disadvantaged caste groups. Ethnicity was significantly associated with the BLL of the children (*p* = 0.001). Among the children with BLL ≥5 μg/dl, 99.4% slept and played in a room containing enamel paint. Of total 201 children who had BLL ≥5 μg/dl, 81.1% had some parts of their house painted with lead containing paints (not shown in table). About one quarter of the children with BLL ≥5 μg/dl had played outside the home while only around one tenth of the children with BLL<5 μg/dl had played outside their home. A large proportion of children with BLL ≥5 μg/dl (42.9%) played with dirt and dust. Only 5.6% of the children with BLL ≥5 μg/dl peeled off paints from the walls of their homes.

**Table 3:** Factors associated with high blood lead levels.

Variables	Blood lead level			*p*-value
	0–5 μg/dl	≥ 5 μg/dl	Total	
**Gender**				0.2
Male	58 (32.8)	119 (67.2)	177 (56.7)	
Female	53 (39.2)	82 (60.7)	135 (43.3)	
**Age groups**				0.1
6–12 months	43 (38.7)	56 (27.9)	99 (33.3)	
13–24 months	46 (41.4)	104 (51.7)	150 (46.6)	
25–36 months	22 (19.8)	41 (20.4)	63 (20.1)	
Ethnic groups*				0.001
Upper caste	59 (54.6)	62 (31.2)	121 (42.9)	
Other ethnic groups	49 (45.4)	137 (68.8)	186 (57.1)	
**Father’s education status**				
Literate	108 (97.3)	194(96.5)	302 (96.8)	0.71
Iliterate	3 (2.7)	7 (3.5)	10 (3.2)	
**Mother’s education status**				
Literate	98 (88.3)	180 (90)	287 (89.4)	0.63
Iliterate	13 (11.7)	20 (10)	33 (10.6)	
**Father’s occupation**				0.2
Agriculture	10 (9.2)	18 (9.0)	28 (9.1)	
Own business	22 (20.2)	26 (13.1)	48 (15.6)	
Service	30 (27.5)	61 (30.7)	91 (29.5)	
Student	10 (9.2)	33 (16.6)	43 (14.0)	
Other	37 (33.9)	61 (30.7)	98 (31.8)	
**Mother’s occupation**				0.4
Housewife	85 (76.6)	148 (74.4)	233 (75.2)	
Agriculture	3 (2.7)	10 (5.0)	13 (4.2)	
Own business	4 (3.6)	11 (5.5)	15 (4.8)	
Service	8 (7.2)	19 (9.5)	27 (8.7)	
Other	11 (9.9)	11 (5.5)	22 (8.7)	
**Household painted with enamel paints**				0.003
Wall	1 (1.1)	18 (11.0)	19 (6.05)	
Window and door	55 (61.8)	64 (39.3)	119(50.55)	
Door and wall	33 (37.1)	81 (49.7)	114 (43.4)	
**School area painted with enamel paint**				0.07
Yes	10 (9.00)	33 (16.4)	43 (12.7)	
No	101 (91.0)	168 (83.6)	269 (87.3)	
**Children sleeping and playing in area with enamel paints**				0.8
Yes	86 (98.9)	161 (99.4)	247 (99.15)	
No	1 (1.1)	1 (0.6)	2 (0.86)	
**Children playing outdoors**				0.003
Yes	12 (10.8)	51 (25.5)	63 (18.1)	
No	99 (89.2)	149 (74.5)	248 (81.8)	
**Children playing with dust and dirt**				0.0001
Yes	24 (21.8)	85 (42.9)	109 (32.3)	
No	86 (78.2)	113 (57.1)	199 (67.6)	
**Children playing with batteries**				0.4
Yes	21 (19.1)	46 (23.1)	67 (21.1)	
No	89 (80.9)	153 (76.9)	242 (78.9)	
**Children playing with painted toys**				0.9
Yes	84 (77.1)	150 (76.9)	234 (77)	
No	25 (22.9)	45 (23.1)	70 (23)	
**Children peeling off paint from walls/windows**				0.1
Yes	2 (1.8)	11 (5.6)	13 (3.7)	
No	107 (98.2)	187 (94.4)	294 (96.3)	

* Information on ethnicity was missing in five cases.

### Relationship between blood lead level and developmental delay in children

We asked parents to report if their child did not acquire a developmental task appropriate for his or her age as compared to peers. The parents of 11.4% of the children with BLL ≥5 μg/dl reported that their children were able to stand and walk later than expected while none of the parents of children with BLL <5 μg/dl reported such developmental delays. However, these findings are limited by the fact that no formal developmental screening or diagnostic tests were performed, and the interviews were taken by persons who had not been specifically trained in developmental assessment.

### Factors affecting blood lead level in multivariable analysis

Table 4 shows factors affecting BLL in multivariate analysis. Multivariate analysis showed that the odds of children playing with dirt and dust to have BLLs ≥5 μg/dl were 4.5 times higher than those of children who did not (OR = 4.53, CI = 1.55–13.20). Similarly, children belonging to other ethnic groups including dalits, disadvantaged janajatis and non-dalit terai caste groups, religious minorities and relatively advantaged janajatis had 3.43 times higher odds of having BLLs ≥5 μg/dl compared to upper caste groups (OR = 3.43, CI = 1.16–10.12).

**Table 4:** Association between blood lead level and variables in bivariate (crude odds ratio) and multivariate models (adjusted odds ratio).

Variable	Crude odds ratio	*p-value*	Adjusted odds ratio	*p-value*
	(95% CI)	* *	(95% CI)	* *
Ethnicity		*** ***		* *
Upper caste ethnic group	1	*0*.*0001*	1	*0*.*02*
Other ethnic groups	2.66 (1.64–4.31)		3.43 (1.16–10.12)	
Children playing outdoors		*** ***		* *
No	1	*0*.*003*	1	*0*.*1*
Yes	2.82 (1.43–5.56)		2.67 (0.74–9.58)	
Children playing in dirt and dust		*** ***		* *
No	1	*0*.*0001*	1	*0*.*006*
Yes	2.69 (1.58–4.59)		4.53 (1.55–13.20)	
Children eating dirt and dust		*** ***		* *
No	1	*0*.*06*	1	*0*.*5*
Yes	2.22 (0.95–5.18)		1.41 (0.44–4.52)	
Schooling of children		*** ***		* *
No	1	*0*.*07*	1	*0*.*07*
Yes	1.98 (0.94–4.20)		3.45 (0.88–13.45)	
Parts of house with enamel paints		*** ***		* *
Door and window only	1	*0*.*001*	1	*0*.*5*
Window door and wall	2.50(1.47–4.25)		1.4 (0.50–3.94)	
Peeling off paints from the wall		*** ***		* *
No	1	*0*.*1*	1	*0*.*8*
Yes	3.15 (0.68–14.46)		1.29 (0.10–16.42)	

## Discussion

Although no child had a BLL >45 μg/dl, which would have required chelation therapy as per CDC guidelines, we found that 64.4% of the children had BLLs exceeding the CDC cut-off point of ≥5 μg/dl indicating a serious public health importance. We chose the age group of 6-36-month-old children because we were interested to enroll pre-school children in our study. However, some children (13.7%) of this age group were found to be already enrolled in pre-school (kindergarten) and we did not exclude these from our study. Comparable results have been obtained in previous studies of primary school children of Birgunj Municipality [17] and Kathmandu Municipality [18], highlighting a continuous health risk in this setting. Such higher elevated blood levels (BLLs ≥5 μg/dl) among children have also been reported in studies conducted in India [19,20]. Similarly, studies conducted in China have also shown a prevalence of elevated BLLs among children, including more than 54% in Jintan [8] and 44% in Wuhan[21]. However, a study conducted in Shanghai revealed a very low prevalence of elevated BLLs among children (<1%) [22] and another one, in Hunan Province, a substantial decline of elevated BLLs from 2009 to 2013 [23]. In our study, BLLs were higher among boys. Higher BLL among boys was also reported in other studies from Nepal [17,18], India [19,20] and China [8,21,22] Although elevated BLLs have been found to be positively correlated with the age of children [8,19,20,22,23] we did not find such association in our study.

The response rate in our study was high (80%) which may be due to counseling parents about the study, providing a free test which is otherwise not available as a regular test in Nepal, and distributing lead test results to parents. We used capillary blood instead of venous blood because obtaining it is less invasive, minimizes pain, requires smaller amounts of blood volume, and the procedure can be performed quickly and easily [24]. Furthermore, it has been found that capillary blood sample collection by fingerstick has very low (<10%) contamination error rates [14,15].

We particularly found higher BLLs in children belonging to socially disadvantaged caste groups. Upper caste groups in Nepal are relatively more economically privileged than the other groups. Ethnic groups such as dalits, disadvantaged janajatis and non-dalit terai caste groups, and relatively advantaged janajatis mostly belong to the lower economic class whereas upper caste groups belong to the higher economic class in Nepal. The socioeconomic status of the family, in turn, can adversely affect the lives of children in many ways. In Nepal as well as in many other countries, poor people usually work in areas with poor hazard control and are often engaged in high-risk labor activities. Children often accompany their parents to such hazardous areas where potential sources of lead may be present, and spend the day playing and sometimes working in that environment without any protection measures. Many of these children even carry food to these places, inadequately wrapped in newspaper, and eat these meals in the same environment without washing their hands. In the USA, the wrapping of sandwiches in newspaper and the behavior of children putting their hand in the mouth have been reported as significant predictors of lead exposure in children [25]. Another study conducted in Michigan, USA, showed that the percentage of children with elevated BLLs increased particularly in socioeconomically disadvantaged neighborhoods after a water source change because of aging water supply infrastructure [26]. In addition, children of certain caste/ethnic groups may also be exposed to higher environmental lead levels due to excessive amounts of dirt and dust in their households. We particularly found higher BLLs in children belonging to socially disadvantaged caste groups. A recent systematic review of the literature also demonstrated a significant effect of ethnicity and racism on BLLs [27]. However, a study conducted in Chitwan valley of Terai region has reported no significant association of cord blood lead level with caste [28].

Some studies found that dust lead levels inside homes were more closely correlated to the BLLs of children than any other source of lead [29,30]; this was also observed in our study. Our findings show that 80.4% of the rooms and houses inhabited by our study participants were painted with enamel paints.

Our results also showed that parental observations of children playing with dirt and dust were significantly associated with higher BLLs. The ingestion of dirt and dust during playtime activity appears to be a more significant pathway for lead uptake by young children than inhalation [31]. Among the children of our study with BLLs ≥5 μg/dl who played in dirt and dust, 39.2% had also been observed by their parents to have eaten dirt and dust. Other investigators have found extensively varying associations between the levels of lead in dust and children's BLLs, with BLLs usually rising by 3–7 μg/dl for every 1,000 ppm increase in dust lead concentration [31–33]. Although playing in dirt and dust was significantly associated with elevated BLLs, factors such as eating dirt and dust and peeling off paint from the wall were not found to be associated with elevated BLLs in our study. This may be partly due to the fact that dirt and dust do not only enter the body through ingestion but also via inhalation and absorption through the skin. Another possibility may be overadjustment in multivariate analysis.

The parents of the children with BLL ≥5 μg/dl reported that their children were able to stand and walk later than expected while none of the parents of children with BLL <5 μg/dl reported such developmental delays in our study. A study conducted in general population of Chitwan valley in Terai region shows significant inverse association of cord blood levels of lead with the neurodevelopment of newborns [5]. However, a recent study by same study group shows prenatal lead levels are not important determinants of the neurodevelopment of less than three years children [34].

As our study was hospital-based and limited to areas within the Kathmandu Valley of Nepal, it does not intend to represent the situation at the national level. However, this study provides important baseline data about BLLs among children in Nepal and associated risk factors. Next, important factors such as breast feeding and food intake of children were not considered in our study. Further studies are needed to determine more nationally representative data and to identify geographic locations with high environmental lead content and factors related to high BLLs of children in Nepal, in order to inform and direct effective public health interventions. The latter should include nation-wide awareness raising campaigns to alert parents and Nepali society as a whole of the lead poisoning that their children are exposed to and its sources. However, as the burden of lead poisoning prevention cannot fall only on parents, the findings of this study also call for further action by policy makers to reduce the concentration of lead in paints and improve the domestic environment of the most affected segments of the population. Implementation of new legislation limiting the concentration of lead in paints and other products requires strict and effective law enforcement, especially in view of the country’s extensive trade across long and porous borders with neighboring India and China. Achieving this, in turn, will critically depend on creating awareness of lead poisoning and related legislation among customs and police forces, and empowering them to enforce it by providing specific training to them.

## Conclusions

Lead is toxic for people of any age group, but young children are at greatest risk because their bodies absorb more of it, and they are more vulnerable than adults to the effects of lead poisoning. Our study revealed that 64.4% of the children aged 6–36 months and living in the Kathmandu Valley of Nepal had high levels of lead in their blood, exceeding the ≥5 μg/dl threshold for specific intervention set by the U.S. Centres for Disease Control and Prevention. Children had higher levels of lead in their blood if enamel paints were used in their house. Higher lead levels were more frequent in children belonging to vulnerable social groups and those who played outdoors with dirt and dust. A study with a nationally representative sample is required to further confirm these findings for populations elsewhere in Nepal, and to identify and validate additional specific public health interventions to reduce the lead exposure of children in Nepal.
