# Correction: High blood levels of lead in children aged 6-36 months in Kathmandu Valley, Nepal: A cross-sectional study of associated factors

**DOI:** 10.1371/journal.pone.0185773

**Published:** 2017-09-28

**Authors:** Meghnath Dhimal, Khem Bahadur Karki, Krishna Kumar Aryal, Bimala Dhimal, Hari Datt Joshi, Sajan Puri, Achyut Raj Pandey, Purushotam Dhakal, Arun Kumar Sharma, Dhruba Shrestha, Ganendra Bhakta Raya, Imran Ansari, David A. Groneberg, Ruth Müller, Ulrich Kuch

Dr. Dhruba Shrestha is not included in the author byline. He should be listed as the tenth author and affiliated with Siddhi Memorial Hospital, Bhaktapur, Nepal. The contributions of this author are as follows: Contributed investigation/project administration/writing—review & editing.
